# Crystal structure of bis­(μ-2-benzoyl­benzoato-κ^2^ *O*:*O*′)bis­[bis­(2,2′-bi­pyridine-κ^2^ *N*,*N*′)manganese(II)] bis­(perchlorate)

**DOI:** 10.1107/S2056989015023671

**Published:** 2015-12-16

**Authors:** Ibrahim Kani

**Affiliations:** a Anadolu University, Faculty of Sciences, Department of Chemistry, 26470 Eskişehir, Turkey

**Keywords:** crystal structure, manganese(II) complex, benzoylbenzoate, 2,2′-bi­pyridine, hydrogen bonding

## Abstract

The title compound, [Mn_2_(C_6_H_5_COC_6_H_4_COO)_2_(C_10_H_8_N_2_)_4_](ClO_4_)_2_, comprises a centrosymmetric binuclear cation and two perchlorate anions. In the complex cation, two Mn^II^ atoms are bridged by two O atoms of two different 2-benzoyl­benzoate ligands, each Mn^II^ atom being further coordinated by two 2,2′-bi­pyridine (bipy) ligands in a distorted octa­hedral environment. Within the binuclear mol­ecule, the Mn⋯Mn separation is 4.513 (7) Å. Inter­molecular C—H⋯O and C—H⋯ π inter­actions link the mol­ecules into a three-dimensional network.

## Related literature   

For applications of inorganic–organic complexes, see: Burd *et al.* (2012 ▸); FitzGerald *et al.* (2013 ▸); Huang *et al.* (2013 ▸); Carrington *et al.* (2014 ▸); Wu *et al.* (2005 ▸); Lee *et al.* (2009 ▸); Li *et al.* (2014 ▸); Zhou *et al.* (2013 ▸); Wang *et al.* (2014 ▸); Hagrman *et al.* (1999 ▸); Ghosh & Bharadwaj (2004 ▸); Evans *et al.* (1999 ▸); Maspoch *et al.* (2007 ▸); Kitagawa & Matsuda (2007 ▸). For manganese complexes with bipyridine, see: Lopes *et al.* (2011 ▸); Knight *et al.* (2010 ▸); McCann *et al.* (1998 ▸); Lumme & Lindell (1988 ▸); Li *et al.* (2002 ▸, 2011 ▸); Wang *et al.* (2012 ▸). 
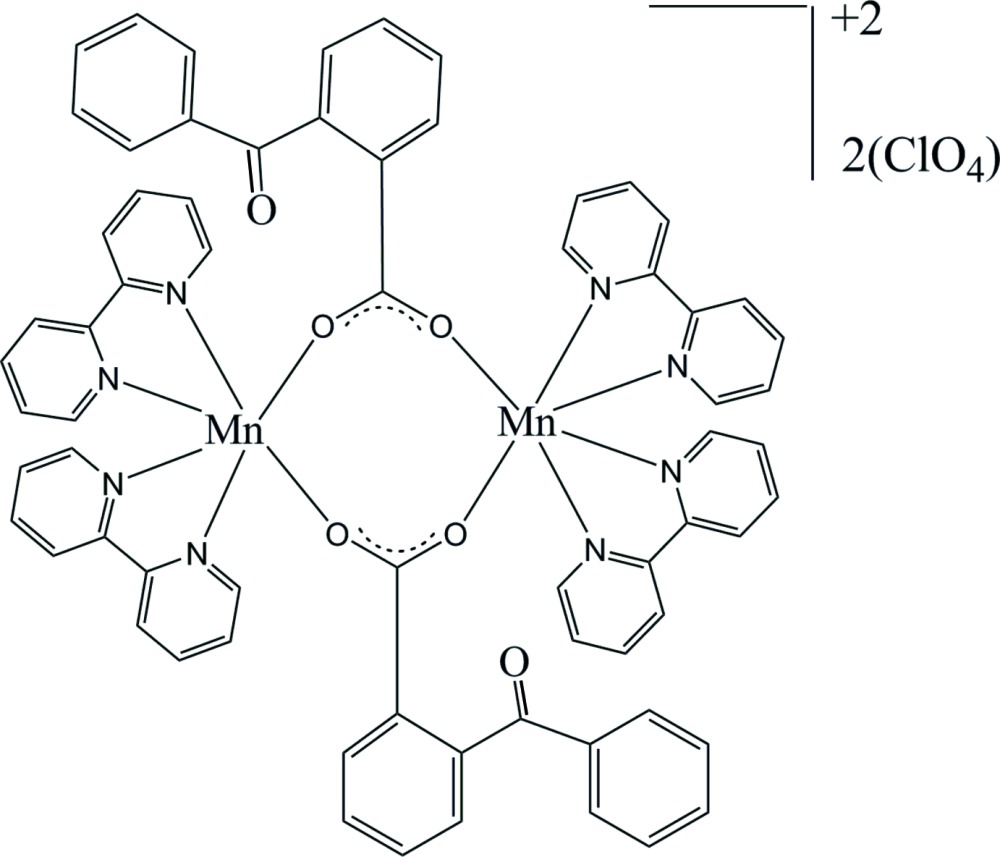


## Experimental   

### Crystal data  

[Mn_2_(C_14_H_9_O_3_)_2_(C_10_H_8_N_2_)_4_](ClO_4_)_2_
*M* *_r_* = 1383.94
Monoclinic, 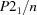

*a* = 13.348 (4) Å
*b* = 17.136 (5) Å
*c* = 14.499 (4) Å
β = 111.321 (10)°
*V* = 3089.3 (16) Å^3^
*Z* = 2
Mo *K*α radiation
μ = 0.57 mm^−1^
*T* = 296 K
0.27 × 0.23 × 0.12 mm

### Data collection  

Bruker APEXII CCD diffractometer
Absorption correction: multi-scan (*SADABS*; Bruker, 2004 ▸) *T*_min_ = 0.857, *T*_max_ = 0.935
39502 measured reflections
7799 independent reflections
5603 reflections with *I* > 2σ(*I*)
*R*_int_ = 0.035

### Refinement  

*R*[*F* ^2^ > 2σ(*F* ^2^)] = 0.039
*wR*(*F* ^2^) = 0.130
*S* = 1.06
6892 reflections
424 parameters
H-atom parameters constrained
Δρ_max_ = 0.52 e Å^−3^
Δρ_min_ = −0.53 e Å^−3^

### 

Data collection: *APEX2* (Bruker, 2007 ▸); cell refinement: *SAINT* (Bruker, 2007 ▸); data reduction: *SAINT*; program(s) used to solve structure: *SHELXS97* (Sheldrick, 2008 ▸); program(s) used to refine structure: *SHELXL97* (Sheldrick, 2008 ▸); molecular graphics: *SHELXTL* ( Sheldrick, 2008 ▸); software used to prepare material for publication: *WinGX* (Farrugia, 2012 ▸).

## Supplementary Material

Crystal structure: contains datablock(s) I. DOI: 10.1107/S2056989015023671/bg2577sup1.cif

**Fig. 1.**
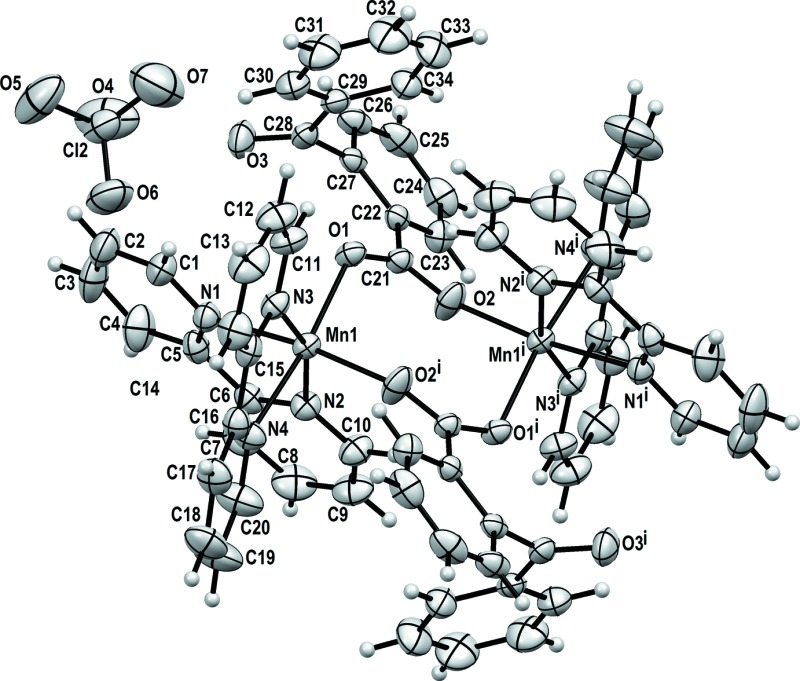
The molecular structure of the title compound, (displacement ellipsoids are shown at 50% probability levels). Symmetry code: (i) −x + 1, −y, −z + 2.

**Fig. 2.**
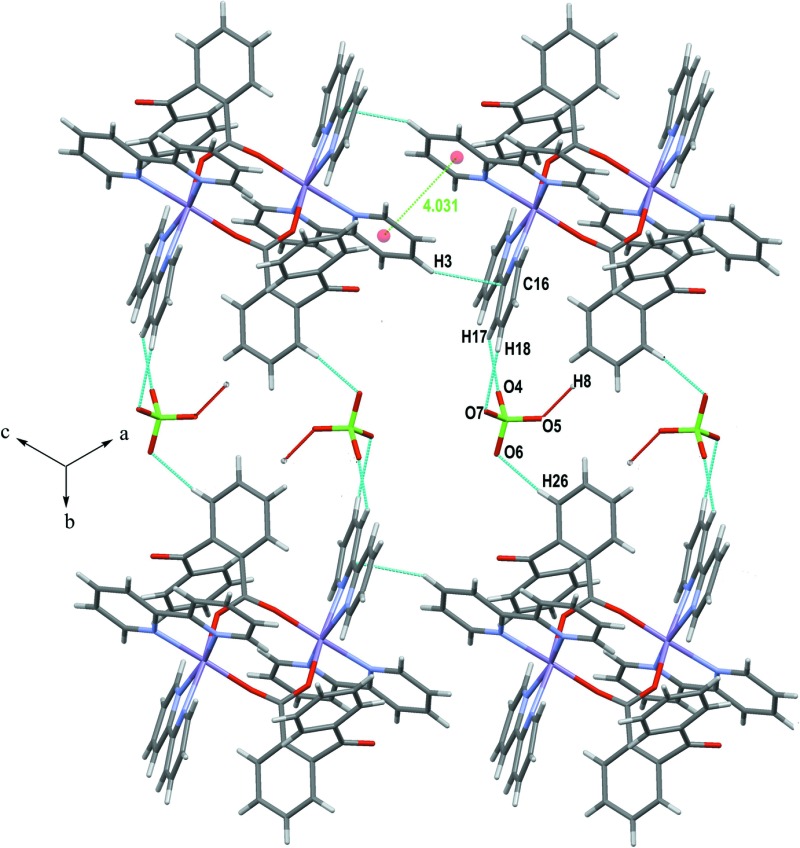
Packing view drawn along the c axis, showing O—H···O, C—H···C hydrogen bonds and C—H··· π, and π–π stacking interactions drawn as dotted lines.

Structure factors: contains datablock(s) I. DOI: 10.1107/S2056989015023671/bg2577Isup2.hkl

x y z . DOI: 10.1107/S2056989015023671/bg2577fig1.tif
The mol­ecular structure of the title compound, (displacement ellipsoids are shown at 50% probability levels). Symmetry code: (i) −*x* + 1, −*y*, −*z* + 2.

c . DOI: 10.1107/S2056989015023671/bg2577fig2.tif
Packing view drawn along the *c* axis, showing O—H⋯O, C—H⋯C hydrogen bonds and C—H⋯ π, and π–π stacking inter­actions drawn as dotted lines.

CCDC reference: 1014518

Additional supporting information:  crystallographic information; 3D view; checkCIF report

## Figures and Tables

**Table 1:** Selected bond lengths (Å)

Mn1—O2	2.0949 (16)
Mn1—O1^i^	2.1260 (14)
Mn1—N3	2.2158 (17)
Mn1—N2	2.2281 (18)
Mn1—N1	2.2555 (18)
Mn1—N4	2.3037 (19)

Symmetry code: (i) 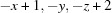.

**Table 2:** Hydrogen-bond geometry (Å, °) *Cg*7 is the centroid of the C22–C27 ring.

*D*—H⋯*A*	*D*—H	H⋯*A*	*D*⋯*A*	*D*—H⋯*A*
C8—H8⋯O5^ii^	0.93	2.65	3.420 (4)	141
C26—H26⋯O6^iii^	0.93	2.58	3.494 (4)	168
C17—H17⋯O4^iv^	0.93	2.46	3.304 (4)	152
C18—H18⋯O7^iv^	0.93	2.72	3.382 (5)	129
C33—H33⋯*Cg*7^iv^	0.93	2.93	3.793 (3)	146

Symmetry codes: (ii) 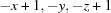; (iii) 
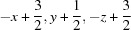
; (iv) 
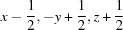
.

## supplementary crystallographic information

### S1. Chemical context

The design of inorganic-organic supra­molecular complexes is of current inter­est in the fields of supra­molecular chemistry and crystal engineering. This inter­est stems from their potential applications as functional materials, such as in gas storage, separation (Burd *et al.*, 2012; FitzGerald *et al.*, 2013; Huang *et al.*, 2013; Carrington *et al.*, 2014), catalysis (Wu *et al.*, 2005; Lee *et al.*, 2009; Li *et al.*, 2014), luminesans, optic, magnetism (Maspoch *et al.*, 2007, Kitagawa &Matsuda 2007, Zhou *et al.*, 2013; Wang *et al.*, 2014), and their further potential medical value derived from their anti­viral and the inhibition of angiogenesis (Hagrman *et al.*, 1999; Ghosh *et al.*, 2004; Evans *et al.*, 1999).

### S2. Structural commentary

In this paper, we will report the synthesis and structure of a new bimetallic manganese complex, [Mn_2_(C_6_H_5_COC_6_H_4_COO)_2_(C_10_H_8_N_2_)_4_](ClO_4_)_2_]. The molecular structure of the complex is illustrated in Fig.1. In the centrosymmetric binuclear molecule the Mn(II) ion is coordinated by two O atoms from two different benzoyl benzoate ligands, four N atoms from two chelating bipy ligands, generating a distorted o­cta­hedral MnN4O2 coordination geometry. The *cisoid* bond angles fall in the region 72.8 (7)–101.5 (7)°, and *transoid* ones are 161.5 (7)°, and 172.9 (7)° exhibiting substantial deviations from an ideal o­cta­hedral geometry.

The Mn–O bond lentghs are 2.095 (2) Å and 2.126 (1) Å (Supplementary Table) The mean Mn—N(bipy) distance of 2.251 (2) Å and the bite angles N1—Mn1—N2 of 73.1 (7)° and N3—Mn1—N3 of 72.8 (4)° are close to the corresponding values observed in related manganese-bipy complexes (Lopes *et al.*, 2011; Knight *et al.*, 2010; McCann *et al.*, 1998; Lumme & Lindell, 1988; Li *et al.*, 2002, 2011; Wang *et al.*, 2012). The dihedral angles between the rings of bipy ligands are −3.8 (3) ° (ligand containing N3 and N4) and −5.6 (3)° (ligand containing N1 and N2).

### S3. Supra­molecular features

In the crystal structure binuclear species are assembled into a three-dimensional supra­molecular architecture by O—H···O, C—H···C hydrogen bonds and C—H··· π, and π–π inter­actions (Fig. 2, Table 2). The closest centroid-centroid distance of the N1,C1—C5 rings is 4.031 Å. The complex molecules are weakly linked by hydrogen bonds through the perchlorate ions to generate the three-dimensional supra­molecular structure.

### S4. Synthesis and crystallization

Mn(ClO_4_)_2_·6H_2_O in methanol (0.076 mmol) was added slowly to a mixed solution of 2,2?-bi­pyridine (0.155 mmol) and benzoyl benzoic acid (0.080 mmol) in methanol (7 ml). After refluxing for 3 h, the mixture was filtered off while hot. The green color single crystals suitable for X-ray analysis were obtained by slow evaporation of the above filtrate at room temperature after a week.

### S5. Refinement

Crystal data, data collection and structure refinement details are summarized in Table 1.

### Crystal data

**Table:**

[Mn_2_(C_14_H_9_O_3_)_2_(C_10_H_8_N_2_)_4_](ClO_4_)_2_	*F*(000) = 1420
*M**_r_* = 1383.94	*D*_x_ = 1.488 Mg m^−^^3^
Monoclinic, *P*2_1_/*n*	Mo *K*α radiation, λ = 0.71073 Å
*a* = 13.348 (4) Å	Cell parameters from 8617 reflections
*b* = 17.136 (5) Å	θ = 2.5–28.2°
*c* = 14.499 (4) Å	µ = 0.57 mm^−^^1^
β = 111.321 (10)°	*T* = 296 K
*V* = 3089.3 (16) Å^3^	Square, yellow
*Z* = 2	0.27 × 0.23 × 0.12 mm

### Data collection

**Table:**

Bruker APEXII CCD diffractometer	7799 independent reflections
Radiation source: fine-focus sealed tube	5603 reflections with *I* > 2σ(*I*)
Graphite monochromator	*R*_int_ = 0.035
phi and ω scans	θ_max_ = 28.5°, θ_min_ = 1.8°
Absorption correction: multi-scan (*SADABS*; Bruker, 2004)	*h* = −16→17
*T*_min_ = 0.857, *T*_max_ = 0.935	*k* = −22→19
39502 measured reflections	*l* = −19→19

### Refinement

**Table:**

Refinement on *F*^2^	Primary atom site location: structure-invariant direct methods
Least-squares matrix: full	Secondary atom site location: difference Fourier map
*R*[*F*^2^ > 2σ(*F*^2^)] = 0.039	Hydrogen site location: inferred from neighbouring sites
*wR*(*F*^2^) = 0.130	H-atom parameters constrained
*S* = 1.06	*w* = 1/[σ^2^(*F*_o_^2^) + (0.073*P*)^2^ + 1.2301*P*] where *P* = (*F*_o_^2^ + 2*F*_c_^2^)/3
6892 reflections	(Δ/σ)_max_ = 0.002
424 parameters	Δρ_max_ = 0.52 e Å^−^^3^
0 restraints	Δρ_min_ = −0.53 e Å^−^^3^

### Special details

**Table:**

Geometry. All e.s.d.'s (except the e.s.d. in the dihedral angle between two l.s. planes) are estimated using the full covariance matrix. The cell e.s.d.'s are taken into account individually in the estimation of e.s.d.'s in distances, angles and torsion angles; correlations between e.s.d.'s in cell parameters are only used when they are defined by crystal symmetry. An approximate (isotropic) treatment of cell e.s.d.'s is used for estimating e.s.d.'s involving l.s. planes.
Refinement. Refinement of *F*^2^ against ALL reflections. The weighted *R*-factor *wR* and goodness of fit *S* are based on *F*^2^, conventional *R*-factors *R* are based on *F*, with *F* set to zero for negative *F*^2^. The threshold expression of *F*^2^ > σ(*F*^2^) is used only for calculating *R*-factors(gt) *etc*. and is not relevant to the choice of reflections for refinement. *R*-factors based on *F*^2^ are statistically about twice as large as those based on *F*, and *R*- factors based on ALL data will be even larger.

### Fractional atomic coordinates and isotropic or equivalent isotropic displacement parameters (Å^2^)

**Table:**

	*x*	*y*	*z*	*U*_iso_*/*U*_eq_	
Mn1	0.33958 (2)	0.022476 (15)	1.01401 (2)	0.03203 (11)	
C29	0.54535 (17)	0.19533 (12)	0.76826 (15)	0.0413 (5)	
Cl2	0.81581 (7)	0.02743 (3)	0.63743 (5)	0.0627 (2)	
O1	0.58328 (12)	0.08588 (7)	0.93327 (10)	0.0384 (3)	
O3	0.72438 (13)	0.22041 (12)	0.87465 (14)	0.0610 (5)	
O2	0.47457 (14)	0.08637 (9)	1.01711 (15)	0.0590 (5)	
O6	0.8226 (2)	−0.04173 (14)	0.6922 (2)	0.0967 (8)	
O5	0.8838 (3)	0.02323 (14)	0.5845 (3)	0.1155 (11)	
O4	0.8379 (3)	0.09348 (15)	0.69834 (19)	0.1176 (11)	
N1	0.18370 (14)	−0.03974 (10)	0.99225 (14)	0.0414 (4)	
N2	0.27238 (15)	−0.01610 (10)	0.85656 (13)	0.0398 (4)	
N3	0.35547 (14)	0.07990 (10)	1.15558 (13)	0.0400 (4)	
N4	0.24957 (16)	0.13976 (10)	0.97526 (14)	0.0472 (4)	
O7	0.7095 (3)	0.0362 (2)	0.5656 (3)	0.1379 (13)	
C5	0.13050 (17)	−0.06874 (12)	0.90196 (17)	0.0437 (5)	
C4	0.0383 (2)	−0.11325 (18)	0.8835 (3)	0.0711 (8)	
H4	0.0008	−0.1328	0.8205	0.085*	
C3	0.0035 (2)	−0.1279 (2)	0.9606 (3)	0.0837 (10)	
H3	−0.0576	−0.1581	0.9499	0.100*	
C2	0.0580 (2)	−0.09852 (18)	1.0515 (3)	0.0691 (8)	
H2	0.0354	−0.1081	1.1039	0.083*	
C1	0.1471 (2)	−0.05439 (15)	1.0643 (2)	0.0532 (6)	
H1	0.1841	−0.0335	1.1267	0.064*	
C6	0.17701 (17)	−0.05233 (12)	0.82610 (16)	0.0421 (5)	
C10	0.3191 (2)	−0.00160 (14)	0.79121 (17)	0.0498 (5)	
H10	0.3855	0.0235	0.8131	0.060*	
C9	0.2740 (3)	−0.02187 (15)	0.69354 (18)	0.0609 (7)	
H9	0.3091	−0.0116	0.6499	0.073*	
C8	0.1752 (3)	−0.05798 (18)	0.6619 (2)	0.0698 (8)	
H8	0.1417	−0.0719	0.5957	0.084*	
C7	0.1266 (2)	−0.07322 (16)	0.72797 (19)	0.0632 (7)	
H7	0.0598	−0.0976	0.7071	0.076*	
C21	0.53450 (15)	0.11944 (10)	0.98092 (14)	0.0343 (4)	
C22	0.55033 (15)	0.20527 (10)	0.99784 (14)	0.0332 (4)	
C27	0.59429 (15)	0.25089 (10)	0.94292 (14)	0.0345 (4)	
C28	0.62929 (17)	0.21976 (11)	0.86348 (16)	0.0405 (5)	
C30	0.5765 (2)	0.15888 (15)	0.69870 (18)	0.0555 (6)	
H30	0.6490	0.1492	0.7118	0.067*	
C31	0.5003 (3)	0.1367 (2)	0.6095 (2)	0.0731 (8)	
H31	0.5215	0.1117	0.5627	0.088*	
C32	0.3929 (3)	0.1511 (2)	0.5893 (2)	0.0808 (9)	
H32	0.3419	0.1354	0.5291	0.097*	
C33	0.3611 (2)	0.1883 (2)	0.6571 (2)	0.0726 (8)	
H33	0.2887	0.1993	0.6426	0.087*	
C34	0.4369 (2)	0.20974 (15)	0.74772 (17)	0.0528 (6)	
H34	0.4152	0.2338	0.7949	0.063*	
C26	0.60483 (18)	0.33052 (12)	0.96073 (18)	0.0482 (5)	
H26	0.6345	0.3616	0.9246	0.058*	
C25	0.5722 (2)	0.36409 (12)	1.0307 (2)	0.0574 (7)	
H25	0.5779	0.4178	1.0403	0.069*	
C24	0.5313 (2)	0.31917 (14)	1.0867 (2)	0.0575 (6)	
H24	0.5107	0.3418	1.1353	0.069*	
C23	0.52106 (18)	0.23995 (13)	1.07001 (17)	0.0458 (5)	
H23	0.4938	0.2092	1.1083	0.055*	
C15	0.32092 (17)	0.15410 (12)	1.15152 (16)	0.0414 (5)	
C16	0.26552 (17)	0.18769 (12)	1.05165 (17)	0.0429 (5)	
C20	0.2014 (3)	0.16810 (16)	0.8851 (2)	0.0727 (8)	
H20	0.1894	0.1346	0.8317	0.087*	
C19	0.1679 (3)	0.24425 (19)	0.8656 (2)	0.0846 (10)	
H19	0.1345	0.2619	0.8009	0.102*	
C18	0.1850 (3)	0.29259 (16)	0.9434 (2)	0.0740 (8)	
H18	0.1639	0.3446	0.9329	0.089*	
C17	0.2335 (2)	0.26480 (14)	1.0375 (2)	0.0582 (6)	
H17	0.2447	0.2975	1.0916	0.070*	
C14	0.3385 (2)	0.19644 (15)	1.23753 (19)	0.0583 (6)	
H14	0.3142	0.2476	1.2344	0.070*	
C13	0.3920 (3)	0.16196 (18)	1.3268 (2)	0.0692 (8)	
H13	0.4037	0.1895	1.3850	0.083*	
C12	0.4285 (3)	0.08675 (17)	1.33073 (19)	0.0643 (7)	
H12	0.4660	0.0627	1.3910	0.077*	
C11	0.4078 (2)	0.04829 (14)	1.24293 (17)	0.0519 (5)	
H11	0.4319	−0.0028	1.2450	0.062*	

### Atomic displacement parameters (Å^2^)

**Table:**

	*U*^11^	*U*^22^	*U*^33^	*U*^12^	*U*^13^	*U*^23^
Mn1	0.0380 (2)	0.02841 (15)	0.03140 (17)	−0.00411 (10)	0.01470 (14)	−0.00338 (10)
C29	0.0429 (12)	0.0437 (10)	0.0358 (10)	−0.0035 (9)	0.0128 (9)	0.0106 (8)
Cl2	0.0910 (5)	0.0447 (3)	0.0708 (4)	0.0073 (3)	0.0514 (4)	0.0037 (3)
O1	0.0505 (8)	0.0298 (6)	0.0389 (7)	−0.0006 (5)	0.0210 (7)	0.0029 (5)
O3	0.0359 (9)	0.0848 (12)	0.0638 (11)	−0.0114 (8)	0.0201 (8)	0.0015 (9)
O2	0.0678 (11)	0.0397 (8)	0.0887 (13)	−0.0165 (7)	0.0512 (10)	−0.0046 (8)
O6	0.128 (2)	0.0668 (13)	0.115 (2)	0.0097 (13)	0.0686 (18)	0.0332 (13)
O5	0.175 (3)	0.0781 (16)	0.152 (3)	−0.0089 (16)	0.129 (2)	−0.0082 (15)
O4	0.210 (3)	0.0697 (14)	0.0783 (16)	0.0250 (18)	0.0579 (19)	−0.0091 (12)
N1	0.0359 (9)	0.0427 (9)	0.0473 (10)	−0.0031 (7)	0.0169 (8)	0.0002 (7)
N2	0.0420 (10)	0.0413 (9)	0.0346 (9)	−0.0044 (7)	0.0120 (8)	−0.0055 (7)
N3	0.0481 (10)	0.0393 (8)	0.0378 (9)	−0.0056 (7)	0.0218 (8)	−0.0049 (7)
N4	0.0536 (11)	0.0409 (9)	0.0429 (10)	0.0056 (8)	0.0126 (9)	−0.0018 (7)
O7	0.120 (3)	0.123 (3)	0.142 (3)	0.0136 (19)	0.013 (2)	0.034 (2)
C5	0.0329 (11)	0.0396 (10)	0.0555 (13)	−0.0022 (8)	0.0125 (10)	−0.0052 (9)
C4	0.0506 (16)	0.0759 (18)	0.086 (2)	−0.0242 (13)	0.0244 (15)	−0.0266 (16)
C3	0.0553 (17)	0.087 (2)	0.122 (3)	−0.0286 (16)	0.0489 (19)	−0.017 (2)
C2	0.0595 (17)	0.0725 (17)	0.090 (2)	−0.0093 (14)	0.0452 (16)	0.0057 (16)
C1	0.0487 (14)	0.0593 (13)	0.0579 (14)	−0.0031 (11)	0.0270 (12)	0.0045 (11)
C6	0.0390 (12)	0.0381 (9)	0.0424 (11)	0.0001 (8)	0.0070 (9)	−0.0054 (8)
C10	0.0581 (15)	0.0540 (12)	0.0395 (12)	−0.0075 (11)	0.0201 (11)	−0.0048 (10)
C9	0.085 (2)	0.0630 (15)	0.0367 (12)	0.0011 (13)	0.0250 (13)	−0.0026 (10)
C8	0.085 (2)	0.0727 (17)	0.0381 (13)	−0.0082 (15)	0.0055 (14)	−0.0109 (12)
C7	0.0603 (17)	0.0682 (16)	0.0460 (14)	−0.0132 (13)	0.0015 (12)	−0.0122 (12)
C21	0.0346 (10)	0.0295 (8)	0.0374 (10)	−0.0036 (7)	0.0114 (8)	0.0032 (7)
C22	0.0283 (10)	0.0300 (8)	0.0379 (10)	−0.0024 (7)	0.0079 (8)	0.0013 (7)
C27	0.0266 (10)	0.0309 (8)	0.0380 (10)	−0.0040 (7)	0.0021 (8)	0.0051 (7)
C28	0.0374 (12)	0.0392 (10)	0.0447 (11)	−0.0051 (8)	0.0148 (9)	0.0096 (8)
C30	0.0577 (15)	0.0670 (15)	0.0441 (13)	0.0021 (12)	0.0212 (12)	0.0096 (11)
C31	0.087 (2)	0.092 (2)	0.0400 (14)	−0.0023 (17)	0.0229 (14)	−0.0025 (13)
C32	0.076 (2)	0.115 (3)	0.0374 (14)	−0.0142 (19)	0.0036 (14)	−0.0023 (15)
C33	0.0487 (16)	0.108 (2)	0.0479 (15)	−0.0024 (15)	0.0013 (13)	0.0014 (15)
C34	0.0447 (14)	0.0673 (14)	0.0421 (12)	−0.0022 (11)	0.0107 (11)	0.0028 (10)
C26	0.0432 (13)	0.0321 (9)	0.0561 (13)	−0.0058 (8)	0.0024 (11)	0.0081 (9)
C25	0.0527 (14)	0.0301 (9)	0.0729 (17)	−0.0001 (9)	0.0031 (13)	−0.0062 (10)
C24	0.0546 (15)	0.0498 (12)	0.0620 (15)	0.0069 (11)	0.0140 (13)	−0.0188 (11)
C23	0.0443 (12)	0.0448 (11)	0.0495 (12)	−0.0028 (9)	0.0185 (10)	−0.0051 (9)
C15	0.0415 (12)	0.0402 (10)	0.0489 (12)	−0.0055 (8)	0.0240 (10)	−0.0084 (8)
C16	0.0404 (12)	0.0387 (10)	0.0523 (12)	−0.0016 (8)	0.0202 (10)	−0.0053 (9)
C20	0.094 (2)	0.0569 (15)	0.0504 (15)	0.0180 (14)	0.0065 (15)	0.0008 (12)
C19	0.103 (3)	0.0684 (18)	0.0653 (19)	0.0278 (17)	0.0095 (18)	0.0171 (15)
C18	0.084 (2)	0.0457 (13)	0.084 (2)	0.0187 (13)	0.0202 (17)	0.0108 (13)
C17	0.0617 (16)	0.0423 (11)	0.0729 (17)	0.0050 (10)	0.0272 (14)	−0.0063 (11)
C14	0.0686 (17)	0.0554 (13)	0.0563 (15)	0.0002 (12)	0.0292 (13)	−0.0174 (11)
C13	0.089 (2)	0.0789 (18)	0.0464 (14)	−0.0084 (15)	0.0320 (14)	−0.0218 (13)
C12	0.083 (2)	0.0711 (17)	0.0387 (13)	−0.0101 (14)	0.0221 (13)	−0.0029 (11)
C11	0.0677 (16)	0.0490 (11)	0.0409 (12)	−0.0048 (11)	0.0221 (11)	0.0031 (9)

### Geometric parameters (Å, º)

**Table:**

Mn1—O2	2.0949 (16)	C8—H8	0.9300
Mn1—O1^i^	2.1260 (14)	C7—H7	0.9300
Mn1—N3	2.2158 (17)	C21—C22	1.493 (2)
Mn1—N2	2.2281 (18)	C22—C23	1.378 (3)
Mn1—N1	2.2555 (18)	C22—C27	1.389 (3)
Mn1—N4	2.3037 (19)	C27—C26	1.386 (3)
C29—C30	1.373 (3)	C27—C28	1.490 (3)
C29—C34	1.390 (3)	C30—C31	1.377 (4)
C29—C28	1.487 (3)	C30—H30	0.9300
Cl2—O5	1.386 (3)	C31—C32	1.377 (5)
Cl2—O4	1.400 (3)	C31—H31	0.9300
Cl2—O6	1.411 (2)	C32—C33	1.364 (5)
Cl2—O7	1.430 (3)	C32—H32	0.9300
O1—C21	1.249 (2)	C33—C34	1.385 (4)
O1—Mn1^i^	2.1260 (14)	C33—H33	0.9300
O3—C28	1.220 (3)	C34—H34	0.9300
O2—C21	1.242 (2)	C26—C25	1.368 (4)
N1—C1	1.329 (3)	C26—H26	0.9300
N1—C5	1.338 (3)	C25—C24	1.368 (4)
N2—C10	1.334 (3)	C25—H25	0.9300
N2—C6	1.339 (3)	C24—C23	1.377 (3)
N3—C11	1.320 (3)	C24—H24	0.9300
N3—C15	1.346 (3)	C23—H23	0.9300
N4—C20	1.322 (3)	C15—C14	1.387 (3)
N4—C16	1.332 (3)	C15—C16	1.482 (3)
C5—C4	1.388 (3)	C16—C17	1.381 (3)
C5—C6	1.473 (3)	C20—C19	1.375 (4)
C4—C3	1.381 (5)	C20—H20	0.9300
C4—H4	0.9300	C19—C18	1.351 (5)
C3—C2	1.349 (5)	C19—H19	0.9300
C3—H3	0.9300	C18—C17	1.366 (4)
C2—C1	1.363 (4)	C18—H18	0.9300
C2—H2	0.9300	C17—H17	0.9300
C1—H1	0.9300	C14—C13	1.364 (4)
C6—C7	1.382 (3)	C14—H14	0.9300
C10—C9	1.367 (3)	C13—C12	1.371 (4)
C10—H10	0.9300	C13—H13	0.9300
C9—C8	1.376 (4)	C12—C11	1.370 (3)
C9—H9	0.9300	C12—H12	0.9300
C8—C7	1.364 (4)	C11—H11	0.9300
			
O2—Mn1—O1^i^	98.53 (7)	O2—C21—C22	117.04 (18)
O2—Mn1—N3	87.49 (7)	O1—C21—C22	118.25 (16)
O1^i^—Mn1—N3	100.61 (6)	C23—C22—C27	119.23 (18)
O2—Mn1—N2	101.52 (7)	C23—C22—C21	119.16 (18)
O1^i^—Mn1—N2	94.08 (6)	C27—C22—C21	121.61 (18)
N3—Mn1—N2	161.48 (7)	C26—C27—C22	118.8 (2)
O2—Mn1—N1	172.97 (7)	C26—C27—C28	117.20 (19)
O1^i^—Mn1—N1	86.51 (6)	C22—C27—C28	123.93 (16)
N3—Mn1—N1	96.45 (7)	O3—C28—C29	121.5 (2)
N2—Mn1—N1	73.10 (7)	O3—C28—C27	119.8 (2)
O2—Mn1—N4	85.27 (7)	C29—C28—C27	118.44 (18)
O1^i^—Mn1—N4	172.33 (6)	C29—C30—C31	119.9 (3)
N3—Mn1—N4	72.80 (7)	C29—C30—H30	120.1
N2—Mn1—N4	91.66 (7)	C31—C30—H30	120.1
N1—Mn1—N4	90.31 (7)	C30—C31—C32	120.5 (3)
C30—C29—C34	119.6 (2)	C30—C31—H31	119.8
C30—C29—C28	118.9 (2)	C32—C31—H31	119.8
C34—C29—C28	121.6 (2)	C33—C32—C31	120.2 (3)
O5—Cl2—O4	111.14 (19)	C33—C32—H32	119.9
O5—Cl2—O6	110.27 (16)	C31—C32—H32	119.9
O4—Cl2—O6	111.62 (18)	C32—C33—C34	119.8 (3)
O5—Cl2—O7	106.2 (2)	C32—C33—H33	120.1
O4—Cl2—O7	107.5 (2)	C34—C33—H33	120.1
O6—Cl2—O7	110.0 (2)	C33—C34—C29	120.1 (2)
C21—O1—Mn1^i^	119.02 (12)	C33—C34—H34	120.0
C21—O2—Mn1	155.61 (17)	C29—C34—H34	120.0
C1—N1—C5	118.9 (2)	C25—C26—C27	120.9 (2)
C1—N1—Mn1	124.31 (16)	C25—C26—H26	119.5
C5—N1—Mn1	116.55 (14)	C27—C26—H26	119.5
C10—N2—C6	119.11 (19)	C24—C25—C26	120.4 (2)
C10—N2—Mn1	123.68 (15)	C24—C25—H25	119.8
C6—N2—Mn1	117.12 (14)	C26—C25—H25	119.8
C11—N3—C15	118.87 (19)	C25—C24—C23	119.1 (2)
C11—N3—Mn1	123.07 (15)	C25—C24—H24	120.4
C15—N3—Mn1	117.58 (14)	C23—C24—H24	120.4
C20—N4—C16	118.0 (2)	C24—C23—C22	121.4 (2)
C20—N4—Mn1	125.78 (17)	C24—C23—H23	119.3
C16—N4—Mn1	115.06 (14)	C22—C23—H23	119.3
N1—C5—C4	120.7 (2)	N3—C15—C14	120.8 (2)
N1—C5—C6	116.23 (18)	N3—C15—C16	116.79 (18)
C4—C5—C6	123.0 (2)	C14—C15—C16	122.4 (2)
C3—C4—C5	118.6 (3)	N4—C16—C17	121.3 (2)
C3—C4—H4	120.7	N4—C16—C15	116.42 (18)
C5—C4—H4	120.7	C17—C16—C15	122.3 (2)
C2—C3—C4	120.1 (3)	N4—C20—C19	123.8 (3)
C2—C3—H3	119.9	N4—C20—H20	118.1
C4—C3—H3	119.9	C19—C20—H20	118.1
C3—C2—C1	118.3 (3)	C18—C19—C20	117.8 (3)
C3—C2—H2	120.8	C18—C19—H19	121.1
C1—C2—H2	120.8	C20—C19—H19	121.1
N1—C1—C2	123.3 (3)	C19—C18—C17	119.7 (2)
N1—C1—H1	118.4	C19—C18—H18	120.1
C2—C1—H1	118.4	C17—C18—H18	120.1
N2—C6—C7	120.6 (2)	C18—C17—C16	119.3 (2)
N2—C6—C5	116.77 (19)	C18—C17—H17	120.3
C7—C6—C5	122.7 (2)	C16—C17—H17	120.3
N2—C10—C9	123.0 (2)	C13—C14—C15	119.0 (2)
N2—C10—H10	118.5	C13—C14—H14	120.5
C9—C10—H10	118.5	C15—C14—H14	120.5
C10—C9—C8	117.9 (3)	C14—C13—C12	120.1 (2)
C10—C9—H9	121.1	C14—C13—H13	119.9
C8—C9—H9	121.1	C12—C13—H13	119.9
C7—C8—C9	119.7 (2)	C11—C12—C13	117.7 (3)
C7—C8—H8	120.2	C11—C12—H12	121.2
C9—C8—H8	120.2	C13—C12—H12	121.2
C8—C7—C6	119.7 (3)	N3—C11—C12	123.5 (2)
C8—C7—H7	120.1	N3—C11—H11	118.2
C6—C7—H7	120.1	C12—C11—H11	118.2
O2—C21—O1	124.70 (17)		

Symmetry code: (i) −*x*+1, −*y*, −*z*+2.

### Hydrogen-bond geometry (Å, º)

Cg7 is the centroid of the C22–C27 ring.

**Table:**

*D*—H···*A*	*D*—H	H···*A*	*D*···*A*	*D*—H···*A*
C8—H8···O5^ii^	0.93	2.65	3.420 (4)	141
C26—H26···O6^iii^	0.93	2.58	3.494 (4)	168
C17—H17···O4^iv^	0.93	2.46	3.304 (4)	152
C18—H18···O7^iv^	0.93	2.72	3.382 (5)	129
C33—H33···*Cg*7^iv^	0.93	2.93	3.793 (3)	146

Symmetry codes: (ii) −*x*+1, −*y*, −*z*+1; (iii) −*x*+3/2, *y*+1/2, −*z*+3/2; (iv) *x*−1/2, −*y*+1/2, *z*+1/2.
