# Supplementary figures and images for: Crystal structure of bis­(μ-2-benzoyl­benzoato-κ2 O:O′)bis­[bis­(2,2′-bi­pyridine-κ2 N,N′)manganese(II)] bis­(perchlorate)
Source: Acta Crystallogr E Crystallogr Commun. 2015 Dec 16;71(Pt 12):m265–6. doi: 10.1107/S2056989015023671 (PMC4719866; doi:10.1107/S2056989015023671)

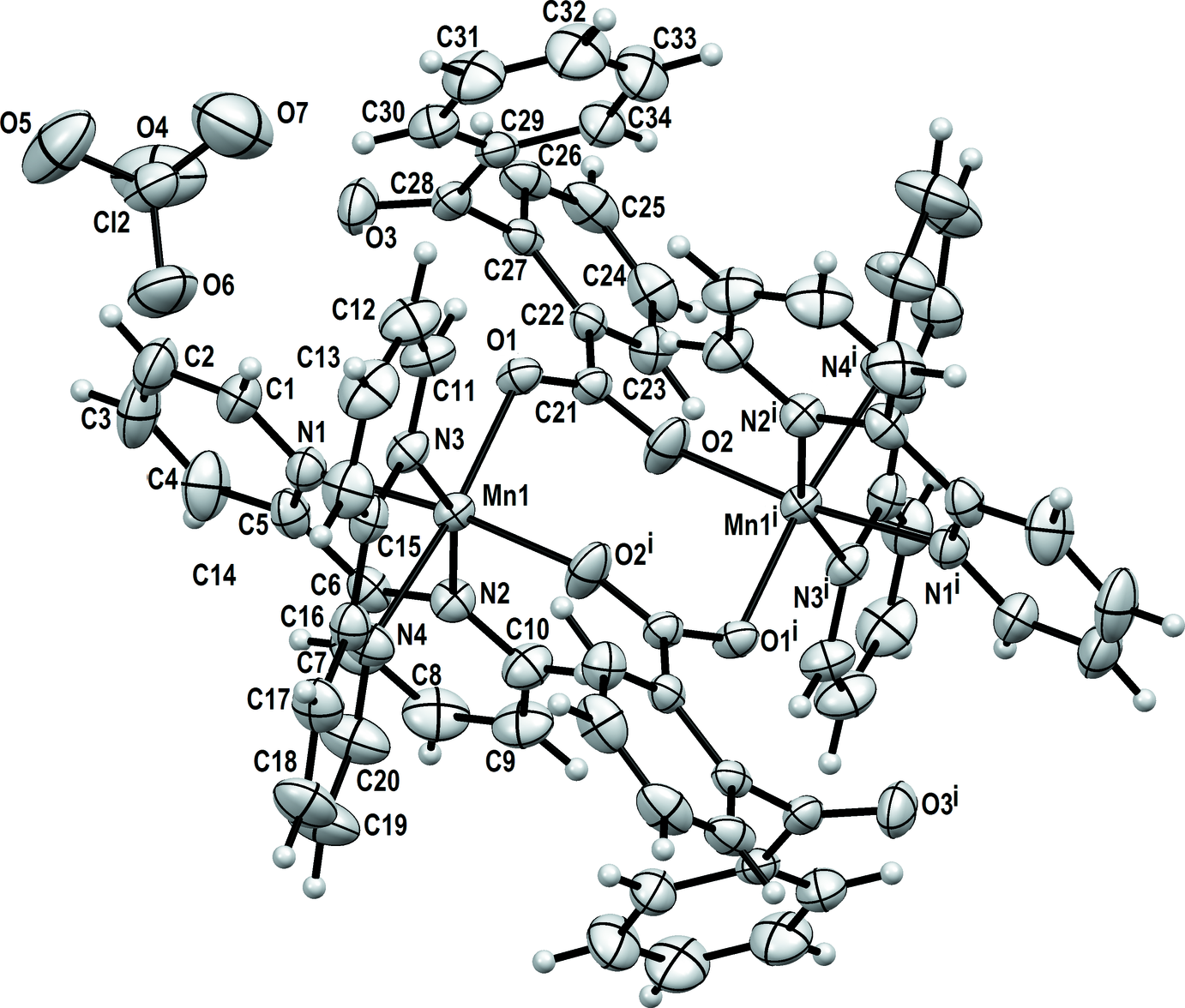

Supplement: Supplementary file 3 [file e-71-0m265-fig1.tif]

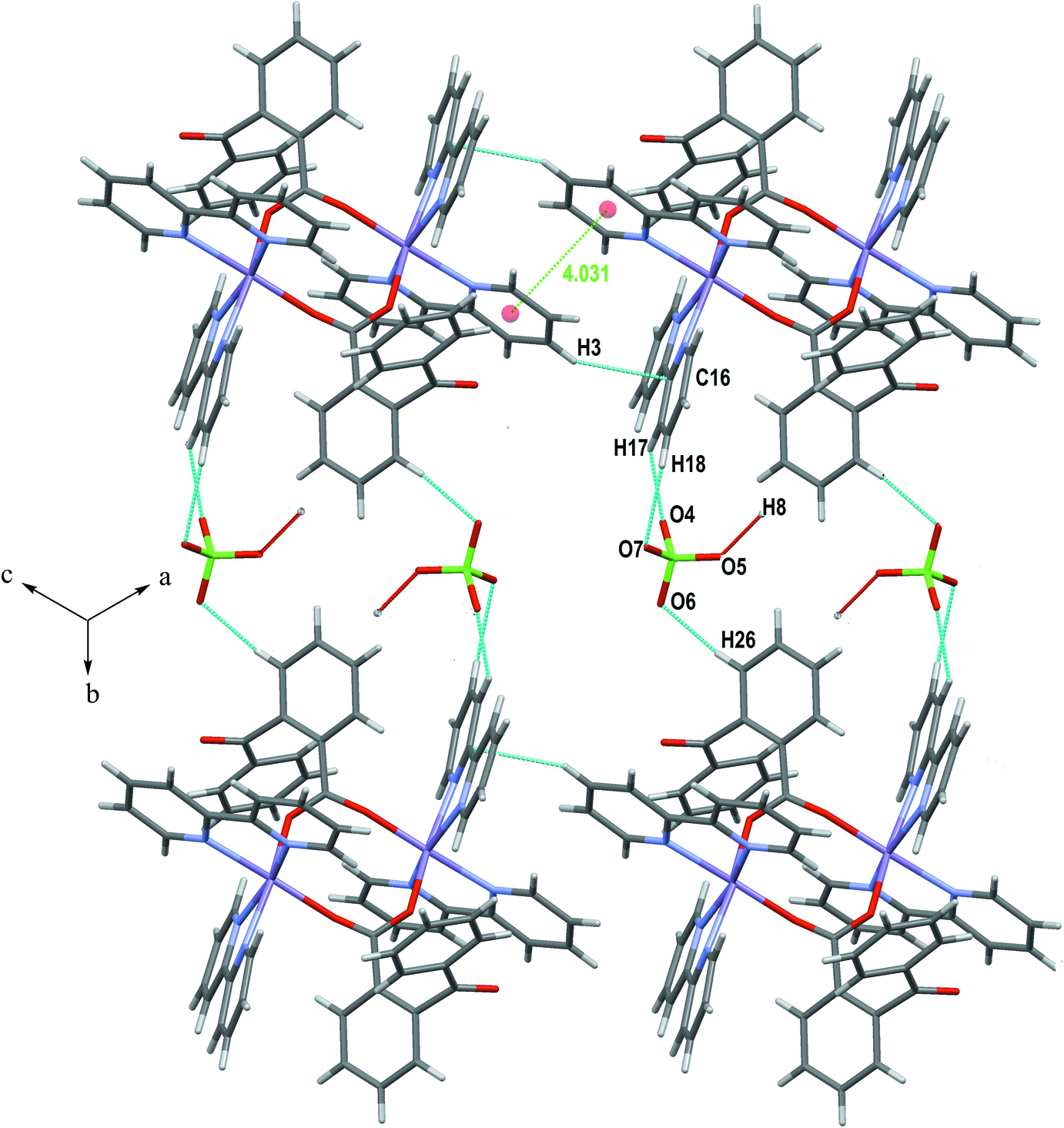

Supplement: Supplementary file 4 [file e-71-0m265-fig2.tif]
